# Corneal Curvature, Astigmatism, and Aberrations in Marfan Syndrome with Lens Subluxation: Evaluation by Pentacam HR System

**DOI:** 10.1038/s41598-018-22358-x

**Published:** 2018-03-06

**Authors:** Jiahui Chen, Qinghe Jing, Yating Tang, Dongjin Qian, Yi Lu, Yongxiang Jiang

**Affiliations:** 1 Department of Ophthalmology and Vision Science, Eye Ear Nose and Throat Hospital of Fudan University, Shanghai, China; 2 Key Laboratory of Myopia of State Health Ministry, and Key Laboratory of Visual Impairment and Restoration of Shanghai, Shanghai, China

## Abstract

Marfan syndrome (MFS) is associated with abnormalities of corneal biometric characteristics. We conducted a retrospective case-control study including 55 eyes of the MFS patients with lens subluxation and 53 normal eyes of the control subjects to evaluate the corneal curvature, astigmatism and aberrations using a rotating Scheimpflug camera (Pentacam HR). Compared with the control group, the anterior, posterior, and total corneal curvature were flatter in the MFS group. The anterior and total corneal astigmatism were higher in the MFS patients, whereas the posterior corneal astigmatism was not significantly different between the two groups. Regarding the total corneal aberrations, the root mean square (RMS) aberrations, RMS higher-order aberrations and RMS lower-order aberrations increased, whereas the spherical aberration decreased in the MFS patients. Corneal parameters had potential diagnostic values for MFS patients with lens subluxation and the more reasonable cutoffs were the values of corneal curvature <41.35 D, corneal astigmatism >0.85 D and spherical aberration <0.188 μm. Corneal biometric characteristics of MFS patients with lens subluxation include decreased corneal curvature, higher corneal astigmatism, larger corneal aberrations, and lower spherical aberration. Corneal curvature, corneal astigmatism, and spherical aberration are better diagnostic tools for suspicious MFS.

## Introduction

Marfan syndrome is a connective tissue disorder, typically caused by mutations in the FBN1 gene, that affects the cardiovascular, skeletal and ocular systems. Aortic root aneurysm, long-bone overgrowth and ectopia lentis (EL) are the cardinal manifestations of MFS^1^. The ocular manifestations of Marfan syndrome include bilateral EL, early myopia, retinal detachment, flat cornea, increased axial length, hypoplastic iris and ciliary muscle hypoplasia^2–4^.

A flat cornea is considered as a diagnostic screening tool for MFS^5–11^; however, there are no reports of anterior or posterior corneal curvature measurement using a Scheimpflug image system. Several studies have indicated that corneal astigmatism, which is an important optical aberration, is higher in MFS patients^8,12^. Both the anterior and posterior corneal surfaces contribute to the total corneal astigmatism (TCA). Therefore, if only the anterior corneal surface is measured, the assessment of the total corneal astigmatism will be imprecise, resulting in an overestimation or underestimation of its value^13–15^. It is clinically important to measure the magnitude of corneal astigmatism accurately in order to calculate the intraocular lens (IOL) power when managing surgery for MFS patients with lens subluxation.

Corneal lower-order aberrations (LOAs) and corneal higher-order aberrations (HOAs) can significantly affect optimal visual acuity^16^. The presence of corneal aberrations can limit the postoperative optical quality after IOL implantation. Although HOAs had been studied many times in refractive surgery, they were rarely reported in MFS patients. The 4th-order spherical aberration (SA), a critical component of HOAs, can be corrected using an aspheric IOL. However, the use of multifocal IOLs is not recommended if there is high irregular corneal astigmatism. On one hand, assessment of corneal HOAs is critical to IOL selection prior to implantation. On the other hand, corneal aberrations measurement may be potential makers for the diagnosis of suspected MFS.

This study investigated the biometric characteristics and diagnostic values of corneal curvature, corneal astigmatism, and corneal aberration measurements in the clinical management of MFS patients with lens subluxation. Use of the Pentacam HR Scheimpflug system, which has greater accuracy than Scheimpflug photography, enabled the simultaneous evaluation of both the anterior and posterior corneal surfaces during patient assessment.

## Results

### Patients demographics

This retrospective case-control study included 108 eyes from 87 subjects who underwent measurements using the Pentacam HR system. The 55 eyes of the 34 MFS patients and the 53 healthy eyes of the 53 control subjects had similar baseline parameters; mean age was 20.5 ± 14.2 years and 22.2 ± 14.2 years, respectively (*P* = 0.459; Mann-Whitney U-test). The demographic characteristics of these two groups are summarized in Table 1.

**Table 1:** Demographic characteristics of the Marfan syndrome and control groups. The data are presented as the means ± standard deviation (SD) and range; MFS = Marfan syndrome.

		MFS	Control	*P* value
Subjects/Eyes		34/55	53/53	
Age (years)	Mean ± SD	20.5 ± 14.2	22.2 ± 14.2	0.459
	Range	4–52	5–53	
Sex (Female:Male)		18:16	19:34	0.116
Eyes (Right:Left)		26:29	30:23	0.332

### Corneal curvature and astigmatism

The corneal curvature (Km value) of the anterior and posterior corneal surfaces was significantly flatter in the MFS group than in the control group (both *P* < 0.001; Student’s *t*-test and Mann-Whitney U-test; Table 2). The total corneal refractive power was also significantly different between the two groups (40.54 ± 1.57 D vs 42.82 ± 1.46 D, *P* < 0.001; Student’s *t*-test). Regarding corneal astigmatism, the anterior corneal astigmatism (ACA) and total corneal astigmatism (TCA) were significantly higher in the MFS group than in the control group (both *P* < 0.001; Mann-Whitney U-test), whereas the posterior corneal astigmatism (PCA) was not significantly different between the two groups. The mean PCA was 0.37 D and exceeded 0.50 D in 14.5% of the MFS eyes.

**Table 2:** Comparison of corneal curvature and corneal astigmatism between the Marfan syndrome and control groups.

		MFS	Control	*P* value
Km F (D)	Mean ± SD	40.90 ± 1.53	43.08 ± 1.45	<**0.001**
	Range	38.5 − 44.0	40.4 − 46.9	
Km B (D)	Mean ± SD	−5.83 ± 0.29	−6.25 ± 0.25	<**0.001**
	Range	−6.4 to −5.0	−6.8 to − 5.8	
TCRP (D)	Mean ± SD	40.54 ± 1.57	42.82 ± 1.46	<**0.001**
	Range	38.1 − 43.9	39.8 − 47.0	
ACA (D)	Mean ± SD	1.75 ± 1.02	0.94 ± 0.69	<**0.001**
	Range	0.2 − 5.4	0.1 − 4.6	
PCA (D)	Mean ± SD	0.37 ± 0.21	0.30 ± 0.16	0.111
	Range	0.0 − 1.0	0.1 − 0.8	
TCA (D)	Mean ± SD	−1.64 ± 0.96	−0.81 ± 0.70	<**0.001**
	Range	−5.4 to − 0.1	−4.5 to –0.1	

The data are presented as the means ± standard deviation (SD) and range. Bold data are significant at *P* < 0.05 (Student’s t-test or Mann-Whitney U-test); MFS = Marfan syndrome; Km = mean keratometry; F = front (anterior corneal surface); B = back (posterior corneal surface); TCRP = total corneal refractive power; ACA = anterior corneal astigmatism; PCA = posterior corneal astigmatism; TCA = total corneal astigmatism; D = diopters.

### Deviation of corneal astigmatism estimated

When the magnitude of ACA (measured in MFS patients and the control group) was used to estimate the TCA, it would overestimate in both groups by a mean of 0.28 ± 0.26 D and 0.23 ± 0.12 D, respectively (Table 3). The distribution of the TCA estimated using ACA measurements alone was not significantly different between the MFS and control groups (*P* = 0.552; Chi-Square test).

**Table 3:** The distribution and magnitude over-, under- and correct estimations of the total corneal astigmatism using the anterior corneal measurements, compared to the total corneal astigmatism measured with Pentacam HR in the Marfan syndrome and control groups.

	MFS group (n = 55)		Control group (n = 53)		*P* value
	n, %	mean ± SD (D)	n, %	mean ± SD (D)	
Overestimation	34, 61.8	0.28 ± 0.26	38, 71.7	0.23 ± 0.12	0.824
Underestimation	11, 20.0	0.33 ± 0.37	8, 15.1	0.23 ± 0.07	0.896
Correct estimation	10, 18.2	/	7, 13.2	/	/

The data are presented as the means ± standard deviation (SD) and percent. MFS = Marfan syndrome; D = diopters.

### Corneal aberrations

**Figure 1:**
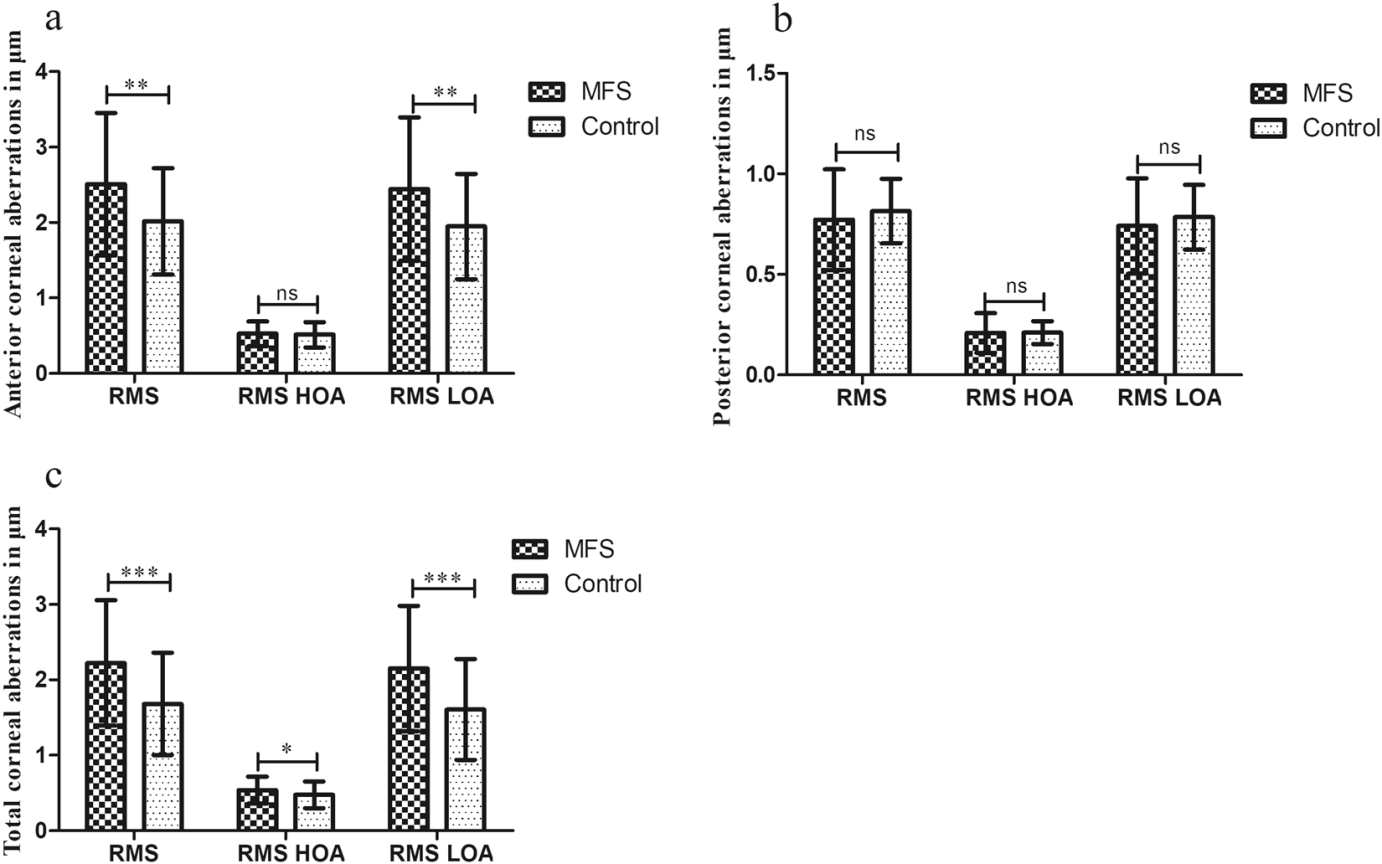
The magnitude of anterior (**a**), posterior (**b**), and total (**c**) corneal aberrations in the Marfan syndrome and control groups. MFS = Marfan syndrome; RMS = root mean square; HOA = higher-order aberration; LOA = lower-order aberration; *P = 0.01 to 0.05; **P < 0.01; ***P < 0.001; ns = not significant.

We analysed the corneal aberrations including the overall RMS, RMS HOA, and RMS LOA for the anterior, posterior, and total cornea (Fig. 1). The RMS (2.507 ± 0.946 μm vs 2.016 ± 0.704 μm, *P* = 0.001; Mann-Whitney U-test) and RMS LOA (2.445 ± 0.949 μm vs 1.948 ± 0.697 μm, *P* = 0.001; Mann-Whitney U-test) of the anterior corneal surface were significantly different between the two groups. There was no significant difference in the posterior corneal aberrations. For the total corneal aberrations, RMS (2.223 ± 0.832 μm vs 1.680 ± 0.677 μm, *P* < 0.001; Mann-Whitney U-test), RMS HOA (0.536 ± 0.178 μm vs 0.474 ± 0.177 μm, *P* = 0.039; Mann-Whitney U-test), and RMS LOA (2.150 ± 0.832 μm vs 1.606 ± 0.668 μm, *P* < 0.001; Student’s *t*-test) were higher in the MFS group than in the control group.

### Higher order aberrations

**Figure 2:**
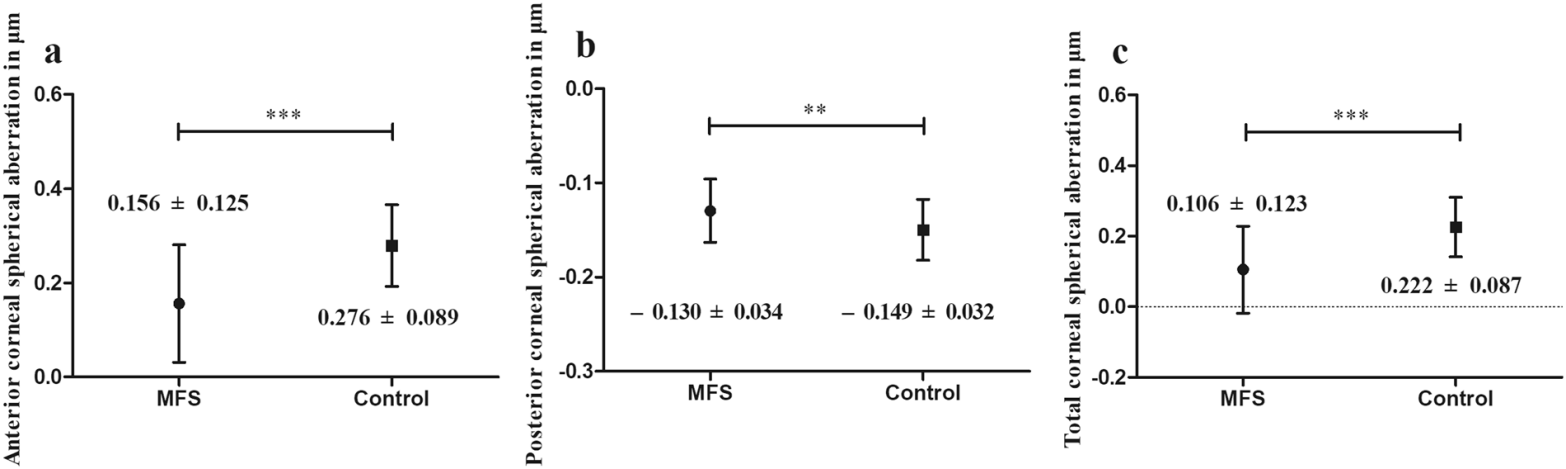
The magnitude of anterior (**a**), posterior (**b**) and total (**c**) corneal spherical aberration in the Marfan syndrome and control groups. MFS = Marfan syndrome; **P < 0.01; ***P < 0.001.

As some 3rd-order aberrations are asymmetry horizontally, we made the separate analysis of the right and left eyes. For the 3rd-order aberrations, there were no significant differences between the MFS and control groups except for the 3rd-order coma, Z(3, −1), of the anterior corneal surface in both the right (*P* = 0.015; Student’s *t*-test) and left eyes (*P* < 0.001; Student’s *t*-test) and total cornea in both the right (*P* = 0.013; Student’s *t*-test) and left eyes (*P* < 0.001; Student’s *t*-test) (Table 4). In the MFS patients, the mean SA values of the anterior corneal surface (0.156 ± 0.125 μm; range, −0.151 to 0.507 μm; *P* < 0.001; Student’s *t*-test), posterior corneal surface (−0.130 ± 0.034 μm; range, −0.268 to −0.075 μm; *P* = 0.002; Student’s *t*-test) and total cornea (0.106 ± 0.123 μm; range, −0.175 to 0.432 μm; *P* < 0.001; Student’s *t*-test) were significantly different compared with the control group (Fig. 2).

**Table 4:** Corneal 3rd-order trefoil and coma aberrations for the Marfan syndrome and control groups.

	Right eye		*P* value	Left eye		*P* value
	MFS (n = 26)	Control (n = 30)		MFS (n = 29)	Control (n = 23)	
Z 3 3 (CF) (μm)	0.006 ± 0.176	−0.023 ± 0.127	0.479	−0.049 ± 0.132	−0.022 ± 0.141	0.483
Z 3 1 (CF) (μm)	−0.093 ± 0.231	−0.169 ± 0.115	0.215	0.131 ± 0.222	0.095 ± 0.127	0.161
Z 3–1 (CF) (μm)	−0.142 ± 0.202	0.003 ± 0.229	**0.015**	−0.177 ± 0.255	0.118 ± 0.309	<**0.001**
Z 3–3 (CF) (μm)	−0.008 ± 0.198	−0.012 ± 0.138	0.706	−0.000 ± 0.195	−0.092 ± 0.203	0.095
Z 3 3 (CB) (μm)	0.005 ± 0.082	0.005 ± 0.054	0.651	0.001 ± 0.113	0.013 ± 0.075	0.513
Z 3 1 (CB) (μm)	−0.008 ± 0.042	0.010 ± 0.019	0.097	−0.005 ± 0.062	0.001 ± 0.029	0.861
Z 3–1 (CB) (μm)	−0.005 ± 0.043	−0.022 ± 0.056	0.209	−0.015 ± 0.049	−0.013 ± 0.065	0.924
Z 3–3 (CB) (μm)	−0.013 ± 0.053	−0.038 ± 0.065	0.133	−0.030 ± 0.122	−0.037 ± 0.101	0.890
Z 3 3 (Cornea) (μm)	0.010 ± 0.206	−0.017 ± 0.152	0.575	−0.049 ± 0.165	−0.012 ± 0.167	0.438
Z 3 1 (Cornea) (μm)	−0.098 ± 0.236	−0.153 ± 0.108	0.440	0.121 ± 0.244	0.089 ± 0.132	0.094
Z 3–1 (Cornea) (μm)	−0.141 ± 0.185	−0.008 ± 0.203	**0.013**	−0.185 ± 0.238	0.117 ± 0.269	<**0.001**
Z 3–3 (Cornea) (μm)	−0.019 ± 0.211	−0.047 ± 0.125	1.000	−0.028 ± 0.226	−0.125 ± 0.237	0.161

The data are presented as the means ± standard deviation (SD). Bold data are significant at *P* < 0.05 (Student’s t-test or Mann−Whitney U-test); MFS = Marfan syndrome; CF = corneal front (i.e. anterior corneal surface); CB = corneal back (i.e. posterior corneal surface).

### Correlations of the corneal curvature

**Figure 3:**
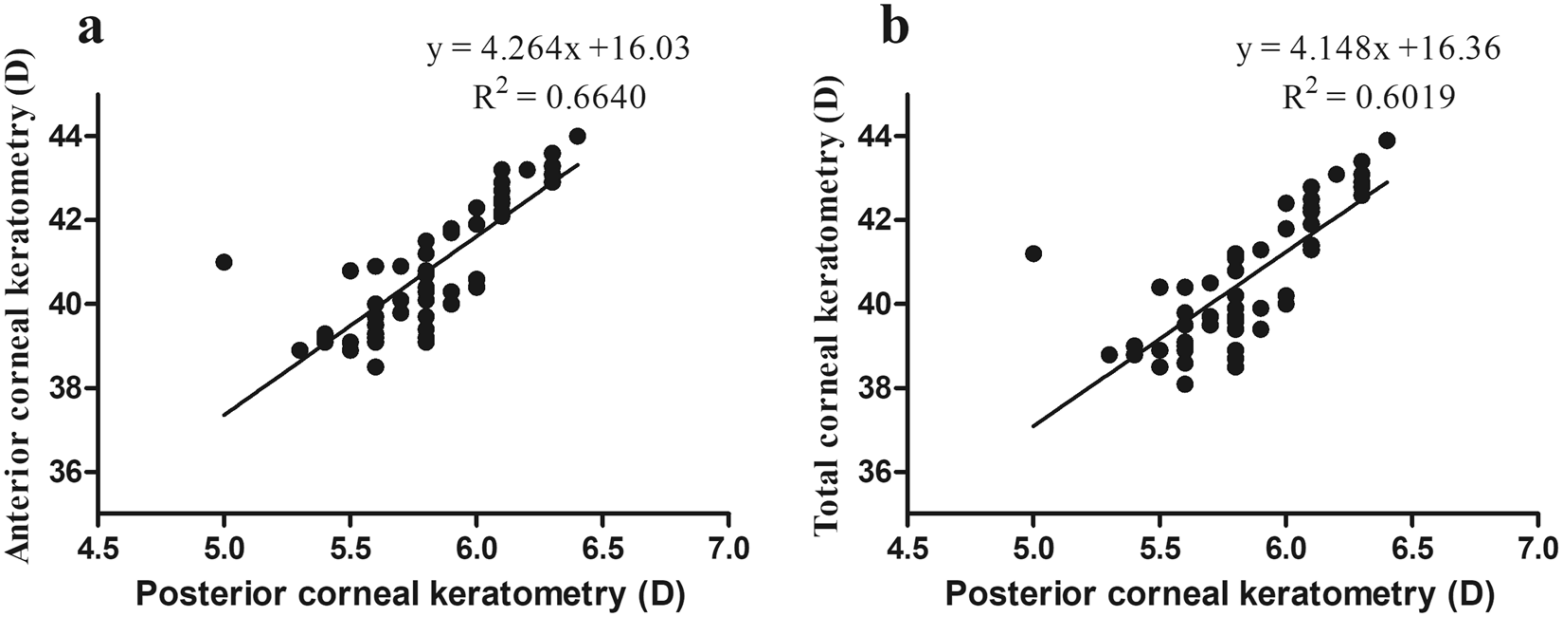
Scatterplots for correlations between the anterior and posterior corneal curvature (**a**), and between the posterior and total corneal curvature (**b**) in the Marfan syndrome. D = diopters.

The corneal curvature was analysed to determine the relative correlations between the posterior corneal surface, the anterior corneal surface and the total cornea in the MFS patients. The Km values were positively correlated between the posterior and anterior corneal surfaces (correlation r = 0.815, *P* < 0.001; Fig. 3a) and between the posterior corneal surface and total corneal refractive power (correlation r = 0.776, *P* < 0.001; Fig. 3b).

### Diagnostic values of corneal parameters

**Figure 4:**
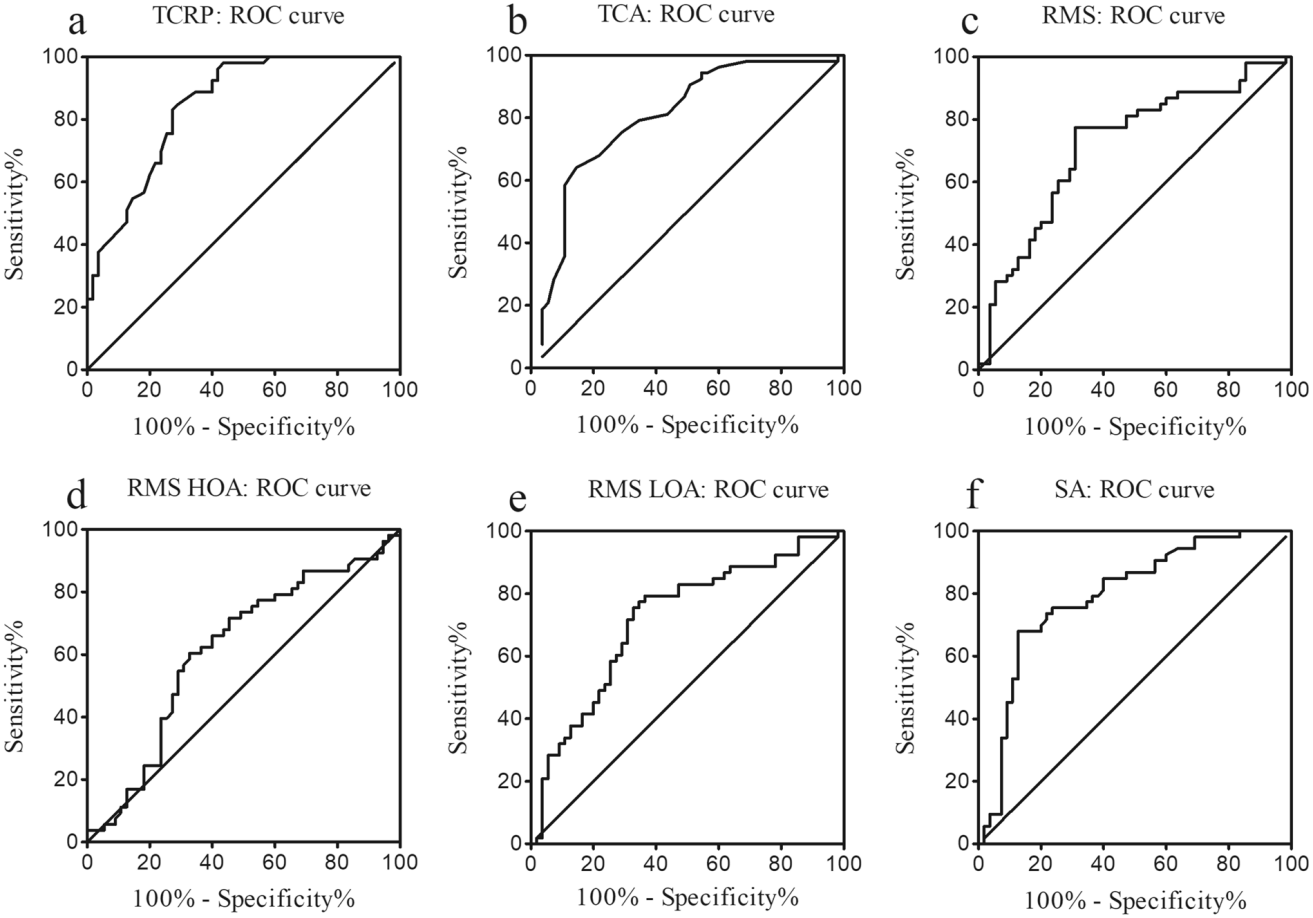
Receiver operating characteristic (ROC) curve for TCRP (**a**), TCA (**b**), RMS (**c**), RMS HOA (**d**), RMS LOA (**e**), and SA (**f**) in relation to the diagnosis of Marfan syndrome. ROC = Receiver operating characteristic; TCRP = total corneal refractive power; TCA = total corneal astigmatism; RMS = root mean square; RMS HOA = RMS of the higher-order aberrations; RMS LOA = RMS of the lower-order aberrations; SA = spherical aberration.

Receiver operating characteristic (ROC) curve was finally analyzed to consider the potential diagnostic values of corneal parameters between the MFS and control groups in Fig. 4. The area under the curve (AUROC) was 0.85 for TCRP, 0.80 for TCA, 0.72 for RMS, 0.62 for RMS HOA, 0.72 for RMS LOA and 0.80 for SA. The value of TCRP at 41.35 D was found to be the optimal cut-off point between the MFS and control groups, which represents a sensitivity of 84.9% and a specificity of 70.9%. Moreover, TCA at −0.85 D, with a specificity of 85.5% and a sensitivity of 64.2%, and SA at 0.188 μm, with a specificity of 87.3% and a sensitivity of 67.9%, indicated good cutoffs between the MFS and control groups. Furthermore, both the anterior and posterior corneal curvature, ACA and spherical aberration showed diagnostic values with the area larger than 0.5 under the curve (0.84 for Km F; 0.86 for Km B; 0.77 for ACA; 0.80 for anterior SA; and 0.72 for posterior SA).

## Discussion

Marfan syndrome is associated with increased axial length, decreased corneal curvature, high corneal astigmatism, and other ocular characteristics^5–9,12^. Corneal characteristics significantly affect the optical quality of the visual image and there is a correlation between the corneal shape and the refractive error and also between the corneal asphericity and the ocular HOAs^17–19^. This study used the Pentacam HR system to assess corneal curvature, corneal astigmatism and corneal aberrations in MFS patients with lens subluxation. Understanding the corneal characteristics of these patients compared with healthy subjects will facilitate earlier diagnosis and more appropriate therapy.

We found a significant difference in the anterior, posterior, and total corneal refractive power between the MFS and control groups. Consistent with previous studies, the corneal curvature was flatter in MFS patients suggesting this as a diagnostic tool for suspected MFS^7–9^. In the present study using the Pentacam HR system, there was a high positive correlation between the posterior corneal curvature and the anterior corneal curvature and also between the posterior corneal curvature and the total corneal refractive power in the MFS group. As the anterior corneal curvature and total corneal refractive power decreased, the posterior corneal curvature decreased, indicating that the cornea was flatter in the MFS patients. For anterior corneal curvature or total corneal refractive power values <41.5 D, the corresponding value of posterior corneal curvature was approximately <6.0 D. Therefore, a posterior corneal curvature value <6.0 D may become an indicator of MFS.

Corneal astigmatism is a visually significant LOA, and MFS patients (with or without lens subluxation) have higher corneal astigmatism^8,12^. Measurements made using the Pentacam HR system in this study revealed higher ACA and TCA values in MFS patients, but no significant difference in PCA values between the MFS and control groups. The mechanism for this difference is unclear. It is proposed that the deviation of EL or the defects in zonular stability and connective corneal tissue due to mutations in the FBN1 gene might increase corneal astigmatism^8^ though PCA was not significantly different between the MFS and control group in the present study. As the anterior corneal surface has a greater role at the corneal refractive interface due to the difference in the refractive index between air and the anterior corneal surface, the smaller difference in the refractive index between the posterior corneal surface and the aqueous humor limits the effect of the PCA^19,20^. Therefore, there was significant difference in ACA but not PCA between the two groups. In addition, only a small cohort of MFS patients with lens subluxation was recruited and further investigation should be conducted to evaluate the significant difference of corneal astigmatism between the MFS with or without lens subluxation and control subjects.

Corneal astigmatism is an important component of overall astigmatic refractive error, and can be corrected by a toric IOL^21^. Their use for the management of lens subluxation in MFS patients decreases the amount of postoperative refractive error and results in more satisfactory surgical outcomes^22^. As both the anterior and posterior corneal surfaces contribute to the TCA, more comprehensive knowledge of the PCA should lead to better optical performance. Although there was no statistically significant difference in PCA between the MFS and control groups (0.37 ± 0.21 D vs 0.30 ± 0.16 D), PCA was higher in MFS patients and therefore should not be ignored. Compared with the measured TCA, the corneal astigmatism calculated by the anterior corneal measurements alone was overestimated by a mean of 0.28 ± 0.26 D and underestimated by a mean of 0.33 ± 0.37 D in the MFS group. In the majority of MFS patients and control subjects, corneal astigmatism was overestimated when only the anterior corneal surface was assessed. Therefore, if the posterior corneal surface cannot be measured, it is very important to underestimate this value before implanting a toric IOL. To accurately calculate the astigmatic correction, the posterior corneal surface measurement should be incorporated into the toric IOL calculation^13,15,23^, especially in MFS patients with high corneal astigmatism.

The ocular aberrations are expressed as the RMS errors and are determined using a series of coefficients for the Zernike terms, point spread functions, modulation transfer functions and other types of metrics^24^. With the Pentacam HR system, a comprehensive analysis is performed to estimate the aberration characteristics and the deterioration of optical quality. An analysis of the corneal aberration differences between MFS patients and healthy subjects has not previously been reported although wavefront analysis has demonstrated that irregular astigmatism exists in normal eyes^24^. In the present study, there were significantly larger values of RMS aberrations and RMS LOAs of the anterior and total cornea in the MFS group than in the control group. However, there were no significant differences in posterior corneal aberrations between the two groups. Different factors such as blinking, accommodation, and age can affect the degree of irregular astigmatism, and the shape and asphericity of the cornea also contribute to increased corneal aberrations^19,24^ which can result in higher anterior and total corneal aberrations in the MFS patients with lens subluxation.

The cornea is the initial component of the visual optics and accounts for approximately 70% of the eye’s refractive power. Furthermore, it is the main contributor to aberrations in the eye^19^. The steeper central region and flatter periphery reduces the amount of SA in the eye^25^. We evaluated the mean SA of the anterior, posterior, and total cornea, using the central 6 mm pupil setting, and found significant differences between the MFS and control groups. The flatter cornea in MFS patients with lens subluxation may be the cause of the lower SA values found in these patients. In MFS patients, the SA should be analyzed prior to implantation of an aspherical IOL, which is designed to compensate for the positive spherical aberration of the cornea.

In receiver operating characteristic curve, many of the corneal parameters were found to have potential diagnostic values for MFS, with a strongest effect being TCRP (AUROC 0.85) and a weakest effect being the RMS HOA (AUROC 0.62). Our results indicated TCRP (cutoff 41.35 D, sensitivity 84.9%, specificity 70.9%) had moderate accuracy for separating the MFS and healthy subjects, which was similar to previous reports^7,9^. Although Luebke J *et al*.^9^ reported Kmax (AUROC 0.82, cutoff 41.36 D) had the strongest effect and Beene LC *et al*.^7^ found a Km value of lower than 41.40 D was the cutoff (AUROC 0.72), it seemed both the Kmax and Km were helpful as screening parameters for MFS. Furthermore, the value of TCA (at −0.85 D, a specificity of 85.5%, a sensitivity of 64.2%), and SA (at 0.188 μm, a specificity of 87.3%, a sensitivity of 67.9%) to predict a diagnosis of MFS were supported in the present study, which had not been reported before. As all the MFS patients recruited had lens subluxation, further study to determine the predictive ability of these parameters to accurately classify MFS patients who did not develop lens subluxations should be conducted to make full use of the diagnostic values of TCRP, TCA, and SA in investigating suspicious MFS.

There are two limitations of this study. Firstly, there is uncertainty regarding the reliability of corneal aberration measurements obtained by the Pentacam HR system. Although previous studies have reported the accuracy of anterior and posterior corneal curvature evaluation (in diopters) by this system^26,27^, no studies have investigated its repeatability in terms of posterior corneal aberrations in MFS patients. Secondly, only a small cohort of patients was recruited and there was no control group of MFS patients without lens subluxation. However, corneal measurements in MFS patients with lens subluxation will contribute to achieving optimum astigmatism and aberration correction for these patients. The results of this study will also contribute to the design of novel IOLs for the treatment of MFS.

In conclusion, the corneal curvature was smaller and the ACA and TCA were higher in the MFS group, whereas PCA was not significantly different between the MFS and control groups. The total corneal aberrations were larger and the SA was lower in the MFS group. Decreased corneal curvature, high corneal astigmatism, and low SA could serve as potential diagnostic tools for patients with suspected MFS with reasonable cutoffs of corneal curvature flatter than 41.35 D, corneal astigmatism higher than 0.85 D and spherical aberration lower than 0.188 μm.

## Methods

### Ethics

A retrospective case-control study was conducted at the Eye and ENT Hospital of Fudan University, Shanghai, China. The medical records of MFS patients treated between July 2015 and May 2017 were reviewed. Written informed consent was obtained from all participants. The study was approved by the Human Research Ethics Committee of the Eye and ENT Hospital of Fudan University, and adhered to the tenets of the Declaration of Helsinki.

### Patient selection

MFS was diagnosed based on the Ghent-2 criteria^28^ and patients with good quality Scheimpflug scans were recruited. The study group comprised 34 MFS patients (55 eyes). The MFS patients had no history of ocular trauma, surgery or other ocular diseases and did not wear contact lenses for 2 weeks prior to Pentacam examination. The control group, matched for age and sex to the MFS group, consisted of 53 subjects (53 eyes) who underwent Pentacam measurements for one normal eye.

### Corneal Assessment

We used the Pentacam HR system (Oculus Inc., Wetzlar, Germany) with a rotating Scheimpflug camera to measure anterior, posterior and total corneal curvature, as well as corneal astigmatism and corneal aberrations. This proprietary method integrates the anterior and posterior corneal surfaces, and the corneal curvature radii are converted into the refractive power using a refractive index of 1.337 for the anterior corneal surface, 1.376 for the posterior corneal surface and 1.336 for the aqueous humor. We measured the following quantitative data: anterior and posterior corneal curvature (mean keratometry, Km), total corneal refractive power (TCRP), anterior corneal astigmatism (ACA), posterior corneal astigmatism (PCA) and total corneal astigmatism (TCA). We also obtained the corneal aberration data. These included the total root mean square (RMS) aberrations, the RMS of the higher-order aberrations (RMS HOA), and the RMS of the lower-order aberrations (RMS LOA) for the anterior, posterior and total corneal surfaces. The following aberration parameters were also recorded: 3rd-order coma Z(3, −1) and Z(3, 1), 3rd-order trefoil Z(3, −3) and Z(3, 3), and 4th-order spherical aberration Z(4, 0) for the anterior, posterior and total corneal surfaces. TCA was calculated using a proprietary method of merging the Scheimpflug data of the anterior and posterior cornea. The corneal aberrations were evaluated using the 6 mm pupil setting based on the elevation data.

### Statistical Analysis

Statistical analyses were performed with SPSS version 20.0 software (IBM Corp., Armonk, NY, USA). Absolute frequency (n) and relative frequency (%) were used to describe qualitative variables. Descriptive statistical results include the mean ± standard deviation (SD) where applicable. The normal distribution of the data was confirmed using the Kolmogorov–Smirnov test. The Student’s *t*-test, Chi-square test, and Wilcoxon rank-sum test (Mann–Whitney *U-*test) were used as appropriate to compare data between the MFS and control groups. The Pearson correlation coefficient was used to evaluate correlations between corneal curvature variables. To assess the diagnostic values of the corneal biometric characteristics, the area under the receiver operating characteristic (AUROC) curves for the whole corneal variable were firstly calculated for separation between the MFS and control groups. For those parameters with an AUROC ≥ 0.80, significant differences of the anterior and posterior corneal surfaces were also tested in their predictive ability to classify MFS patients. A *P* value of < 0.05 was considered significant.

### Data Availability

All data generated or analyzed during this study are included in this published article.
